# Dietary Inulin Fibers Prevent Proton-Pump Inhibitor (PPI)-Induced Hypocalcemia in Mice

**DOI:** 10.1371/journal.pone.0138881

**Published:** 2015-09-23

**Authors:** Mark W. Hess, Jeroen H. F. de Baaij, Lisanne M. M. Gommers, Joost G. J. Hoenderop, René J. M. Bindels

**Affiliations:** Department of Physiology, Radboud Institute for Molecular Life Sciences, Radboud university medical center, Nijmegen, The Netherlands; Wageningen University, NETHERLANDS

## Abstract

**Background:**

Proton-pump inhibitor-induced hypomagnesemia (PPIH) is the most recognized side effect of proton-pump inhibitors (PPIs). Additionally, PPIH is associated with hypocalcemia and hypokalemia. It is hypothesized that PPIs reduce epithelial proton secretion and thereby increase the pH in the colon, which may explain the reduced absorption of and Mg^2+^ and Ca^2+^. Fermentation of dietary oligofructose-enriched inulin fibers by the microflora leads to acidification of the intestinal lumen and by this enhances mineral uptake. This study aimed, therefore, to improve mineral absorption by application of dietary inulin to counteract PPIH.

**Methods:**

Here, C57BL/J6 mice were supplemented with omeprazole and/or inulin. Subsequently, Mg^2+^ and Ca^2+^ homeostasis was assessed by means of serum, urine and fecal electrolyte measurements. Moreover, the mRNA levels of magnesiotropic and calciotropic genes were examined in the large intestine and kidney by real-time PCR.

**Results:**

Treatment with omeprazole significantly reduced serum Mg^2+^ and Ca^2+^ levels. However, concomitant addition of dietary inulin fibers normalized serum Ca^2+^ but not serum Mg^2+^ concentrations. Inulin abolished enhanced expression of *Trpv6* and *S100g* in the colon by omeprazole. Additionally, intestinal and renal mRNA levels of the *Trpm6* gene were reduced after inulin intake.

**Conclusions:**

This study suggests that dietary inulin counteracts reduced intestinal Ca^2+^ absorption upon PPI treatment. In contrast, inulin did not increase intestinal absorption of Mg^2+^ sufficiently to recover serum Mg^2+^. The clinical potential of dietary inulin treatment should be the subject of future studies.

## Introduction

Since their introduction two decades ago, proton pump inhibitors (PPIs) became the mainstay in gastro esophageal reflux (GERD), peptic ulcer disease (PUD), persistent non-steroidal anti-inflammatory drug (NSAID) treatment and generalized dyspepsia (heartburn) [1–3]. PPIs form a class of drugs that is widely prescribed, with millions of chronic users worldwide. [4]. The most recognized side effect of all marketed PPIs is proton-pump inhibitor-induced hypomagnesemia (PPIH) [5, 6]. First described in 2006, thereafter many single case-reports and small case-series on the subject emerged [7–9]. The U.S. Food- and Drug Administration (FDA) issued a warning in 2011 and the critical assessment of their adverse events databank showed that more cases existed than was previously assumed [10]. It is widely anticipated that PPIH is the consequence of intestinal Mg^2+^ malabsorption, since a renal leak was never detected [6, 11]. An underappreciated aspect of PPIH is frequent secondary electrolyte disturbances such as hypocalcemia and hypokalemia [8, 12, 13]. However, the clinical significance of reduced calcium (Ca^2+^) levels was emphasized by several dozens of studies showing increased risk of bone fractures after chronic PPI use [14].

The exact mechanism by which PPIs cause mineral deficits is currently under debate. Recent *in vitro* data suggest that omeprazole inhibits passive paracellular Mg^2+^ fluxes, predominantly present in the small intestine [15, 16]. Moreover, *in vivo* studies indicate that omeprazole directly interferes with important transcellular Mg^2+^ transport mechanisms of the colon [17]. In the tight epithelium of colon, the epithelial Mg^2+^ channel, transient receptor potential melastatin member 6 (TRPM6), facilitates the absorption of Mg^2+^ [18–20]. Omeprazole specifically enhances the mRNA levels of *Trpm6* as well as *Atp12a*, which encodes the non-gastric proton potassium adenosine triphosphatase (colonic H^+^,K^+^-ATPase or cHK-α) [17]. It has been suggested that omeprazole locally inhibits the cHK-α, leading to an increased luminal pH in the colon [17]. Given that TRPM6-mediated Mg^2+^ transport is dependent on the protonation of the channel itself, PPI-induced pH increases will reduce TRPM6-mediated Mg^2+^ influx [21–23].

To date, defined intervention strategies preventing PPIH have not been established in clinical practice. Local luminal acidification of the colon may rescue intestinal Mg^2+^ absorption and, therefore, provides a promising approach to prevent PPIH. Interestingly, the dietary application of the fructan fiber inulin has been proposed to reduce intestinal pH [24]. Ingested inulin fibers are fermented in the large intestine by bifidogenic gut bacteria, resulting in short-chain fatty acids (SCFA), which in turn acidify the colon [25]. The stimulating action of SCFA on intestinal Mg^2+^ absorption by reducing the luminal pH has already been described decades ago, but has been largely overlooked since then [26, 27]. However, inulin fibers have been shown to stimulate Mg^2+^ and Ca^2+^ absorption in the colon of mice and humans [28, 29]. Moreover, inulin fibers are capable of modulating intestinal and renal *Trpm6* mRNA expression [30].

Here, an intervention study was performed using dietary oligfructose-enriched inulin fibers in control- and omeprazole-treated wildtype C57BL/6J male mice. The aim was to enhance intestinal Mg^2+^ and Ca^2+^ absorption in order to counteract omeprazole-induced defects in mineral uptake. Additionally, the mRNA expression pattern of the Mg^2+^ and Ca^2+^ transporting proteins expressed in the cecum, colon and kidney was determined by qRT-PCR.

## Materials and Methods

### Animal studies

This study was carried out in strict compliance with the legal Dutch animal welfare act. All experimental procedures were approved by the animal ethics board of the Radboud University Nijmegen (permit-no: RU-DEC 2014–032) and all efforts were made to minimize suffering of the animals. Wild-type C57BL/6J mice (n = 40 males, 9 weeks old) were purchased from Charles River, the Netherlands. The animals were randomly allocated into four experimental groups of n = 10 mice. Before the experiment was started, the animals underwent acclimatization for one week under temperature- and light-controlled conditions with *ad-libitum* access to standard pellet chow (SSNIFF Spezialdiäten GmbH, Germany) and drinking water. The control diet consisted of standard pellet chow, the experimental inulin diet additionally contained 10% (w/w) inulin fiber product (Orafti Synergy1, Beneo-Orafti, Belgium). Omeprazole (Fagron, the Netherlands) was dispersed in a solution (vehicle) containing 0.5% (w/v) methylcellulose and 0.2% (w/v) NaHCO_3_ (adjusted with NaOH to pH 9.0). During the 14 days of the experimental phase, the mice received a daily dose of 20 mg omeprazole per kilogram bodyweight, administered via oral gavage once a day, or vehicle. The health of the animals was regularly checked. For urine and feces collection, animals were housed individually in metabolic cages for 24 h. At the experimental endpoint, animals were anesthetized with isoflurane (5% v/v), blood sampling was performed by orbital sinus bleeding and subsequently the mice were sacrificed by cervical dislocation. Kidneys, cecum and colon segments were extracted and cleaned, fecal contents of cecum and colon were preserved and all samples were snap frozen in liquid nitrogen.

### Analytical procedures

Serum Mg^2+^, Ca^2+^, K^+^ and Na^+^ concentrations were determined at the university hospital central clinical lab on an automated system according to the manufacturer's protocol (Abbott Diagnostics, Belgium). Feces were homogenized and digested in nitric acid (concentrated with 65% (w/w) Sigma-Aldrich, USA) by a 2 h pre-incubation at 50°C, followed by an overnight incubation at room temperature. Urinary and fecal Mg^2+^ concentrations were determined with a colorimetric xylidyl-II blue kit (Cobas Roche Diagnostics, UK) on a Nanodrop 2000c spectrophotometer (Thermo Fisher Scientific, USA) at 600 nm wavelength. Urinary and fecal Ca^2+^ concentrations were spectrometrically determined with a colorimetric chromogenic/buffer dual-component kit (Sigma Aldrich, UK) on a Biorad plate reader (Biorad, USA) at 570 nm wavelength. The obtained values for Mg^2+^ and Ca^2+^ were cross-verified using a serum standard solution (Precinorm U, Roche, Switzerland).

The SCFA profile of cecal and colonic contents was determined on a Chrompack Model CP 9001 gas chromatograph (Agilent, USA) equipped with a 2 m x 2 mm column, packed with 10% SP 1200/1% H2PO4 on 80/100 Chromosorb W AW (Sigma, UK). Samples were centrifuged at 15,000 x g for 10 min. Subsequently fecal water (supernatant) was extracted and 1:1 (v/v) diluted with a solution (internal standard) of 30 mmol/L of 2-ethylbutyric acid in 100% formic acid, resulting in a 9% (v/v) formic acid suspension used for injection into the gas chromatograph.

### Quantitative real-time PCR

Total RNA was extracted from tissues using TRIzol reagent (Invitrogen, UK) according to the manufacturer’s protocol. Obtained RNA was subjected to DNase treatment (Promega, USA). Subsequently, the purified RNA was reverse transcribed with murine leukemia virus reverse transcriptase (Invitrogen, the Netherlands).

The mRNA expression was quantified by SYBR Green (BioRad, USA) real-time PCR on a CFX96 real-time detection system (BioRad, USA). Real-time PCR primers (Biolegio, the Netherlands) were designed with Primer 3 software (Whitehead Institute for Biomedical Research, USA). Primer sequences are provided in S1 Table. Obtained mRNA levels were normalized by glyceraldehyde 3-phosphate dehydrogenase (*Gapdh*) as an endogenous control. Relative mRNA expression was analyzed according to the Livak method (2^-ΔΔcT^) and annotated as times-fold change of expression compared to control [31].

### Statistics

Values are expressed as means ± SEM. The differences between single groups of control, inulin-only, omeprazole-only and omeprazole + inulin treated mice were tested by using one-way ANOVA with a Tuckey correction. Differences between groups were regarded to be statistically significant when *P < 0*.*05*. The analysis of the datasets was performed using GraphPad Prism (PC version 6).

## Results

### Omeprazole treatment results in reduced serum Mg^2+^

In this study, we aimed to evaluate the application of dietary oligofructose-enriched inulin fibers as a means to prevent PPIH. Therefore, wildtype C57BL6/J mice were supplemented with vehicle or omeprazole for 14 days. In addition, the vehicle group and the omeprazole group were subdivided into two groups fed with a normal diet or with a diet containing 10% w/w oligofructose-enriched inulin fructan fibers, making a total of four experimental groups (Table 1). After 14 days of experimental procedure, the mean bodyweight of the animals was equal (Table 1). Compared to the control group, food intake, water intake and diuresis did not change due to omeprazole or inulin treatment. The mean fecal output of the inulin-omeprazole-treated group was significantly elevated compared to mice receiving only omeprazole (1.5 ± 0.2 g and 1.05 ± 0.06 g, respectively, *P* < 0.05).

**Table 1:** Metabolic parameters of the animals.

	Control		Inulin		Omeprazole		Inulin-Omeprazole	
Diet	normal		inulin		normal		inulin	
Omeprazole treatment	vehicle		vehicle		omperazole		omeprazole	
	Mean	SEM	Mean	SEM	Mean	SEM	Mean	SEM
Body weight (g)	23.8	0.4	24.2	0.5	24.0	0.4	23.7	0.4
Food intake (g)	3.8	0.3	4.2	0.2	3.2^#^	0.2	4.2	0.2
Fecal dry weight (g/24h)	1.13	0.06	1.26	0.05	1.05	0.06	1.5^#^	0.2
Water intake (mL/24h)	3.9	0.4	4.1	0.1	3.9	0.4	4.1	0.4
Diuresis (mL/24h)	1.30	0.07	1.38	0.10	1.02	0.11	1.23	0.08

The results are expressed as means ± SEM (n = 10). Significant differences between the two omeprazole-treated groups are indicated by

^#^ with *P* < 0.05. Mice have been fed a control diet or a diet containing 10% (w/w) oligofructose enriched inulin fibers. Treated mice received 20 mg/kg bodyweight omeprazole.

**Fig 1:**
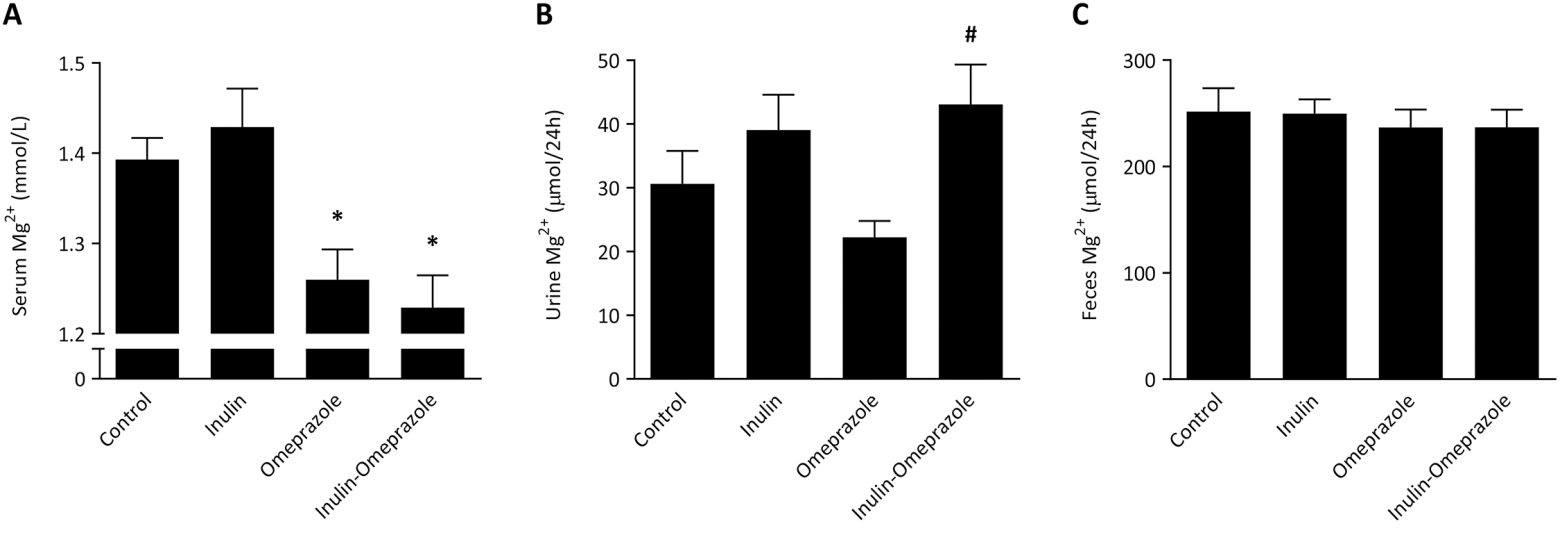
Effects of inulin diets and omeprazole treatment on the Mg^2+^ homeostasis of C57BL/6J mice. Serum Mg^2+^ concentration (A), 24 h urinary Mg^2+^ excretion (B) and 24 h fecal Mg^2+^ excretion (C). All values are presented as means ± SEM (n = 10 per group). Significant differences between control vs. both omeprazole groups are indicated by * with *P* < 0.05, significant differences between the omeprazole-treated groups are annotated by # with *P* < 0.05.

To study the effect of omeprazole and a possible combinatory effect of the inulin diet on Mg^2+^ homeostasis, serum Mg^2+^ concentration, 24 h urinary Mg^2+^ and fecal Mg^2+^ excretion were determined. Omeprazole-treated and inulin-omeprazole-treated mice had significantly lower serum Mg^2+^ levels compared to control mice ((1.26 ± 0.03 mmol/L, 1.23 ± 0.04 mmol/L) and 1.39 ± 0.02 mmol/L, respectively, *P* < 0.05 Fig 1A). Urinary Mg^2+^ excretion was significantly increased in the inulin-omeprazole group compared to omeprazole-treated group (1.0 ± 0.1 mmol/L and 1.23 ± 0.08 mmol/L, respectively, *Ρ* < 0.05 Fig 1B). Fecal Mg^2+^ excretion was equal among all groups (Fig 1C).

### Dietary inulin reduces the expression of *Trpm6*

**Fig 2:**
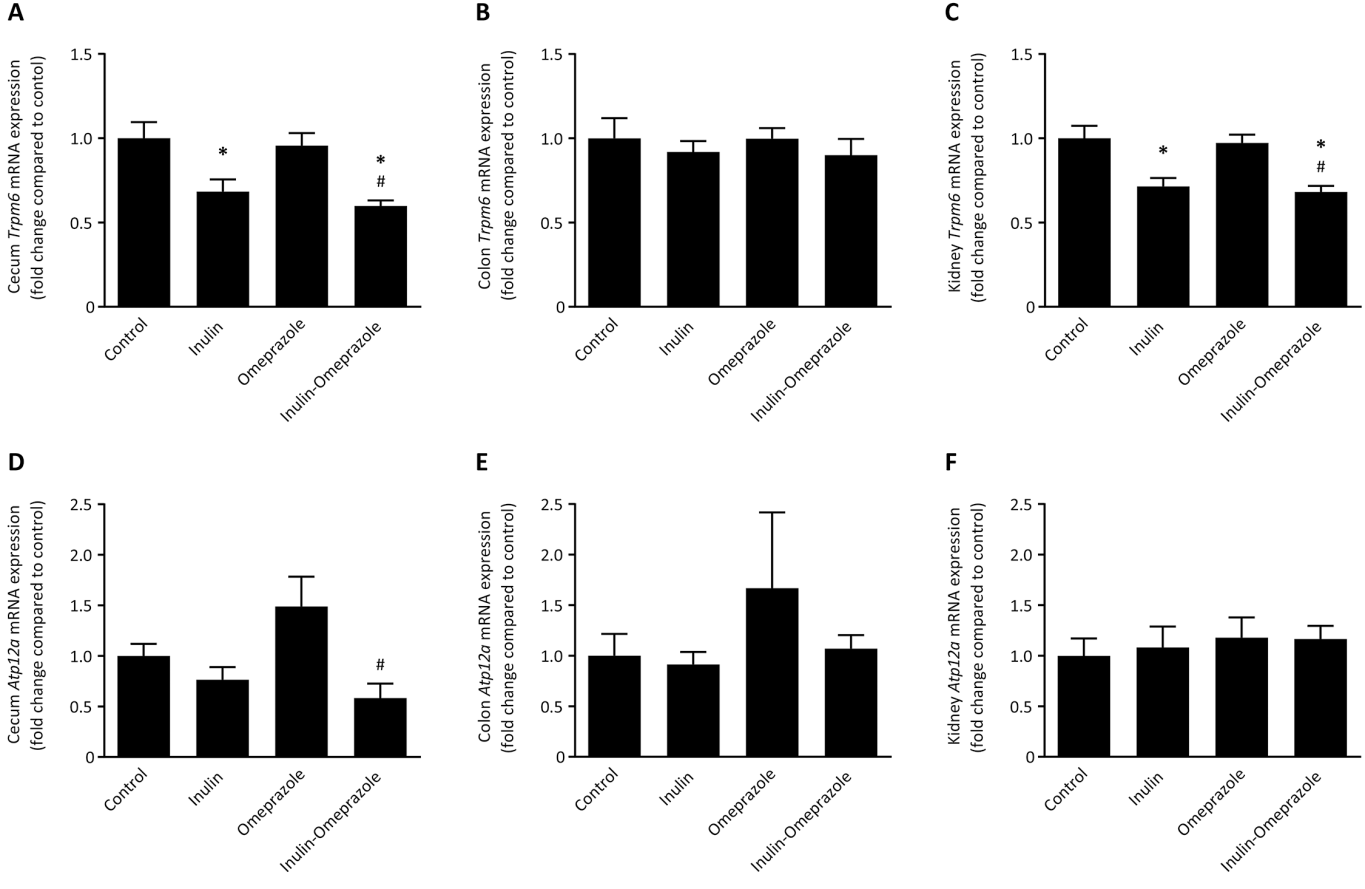
Effects of omeprazole treatment and diets enriched with 10% (w/w) inulin on mRNA levels of PPIH candidate genes. Expressional levels of *Trpm6* and *Atp12a*, encoding the nongastric H^+^,K^+^-ATPase in cecum (A + D), colon (B + E) and kidney (C + F) of C57BL/6J mice compared to control (corrected for *Gapdh* expression). Significant differences compared to control are indicated by * with *P* < 0.05, significant differences between the omeprazole-treated groups are annotated by # with *P* < 0.05. All values represent means ± SEM, with n = 10 mice per group.

To identify the effect of omeprazole treatment and inulin-enriched diets, the mRNA levels of genes involved in Mg^2+^ transport were evaluated. In cecum, addition of inulin to the diet significantly reduced *Trpm6* mRNA levels by 32 ± 7% compared to vehicle-treated mice on the normal diet and by 40 ± 3% in mice that received inulin combined with omeprazole (Fig 2A). In colon, the T*rpm6* mRNA levels were not significantly different between the groups (Fig 2B). Accordingly, *Trpm6* mRNA levels were reduced by 29 ± 5% in the inulin group compared to the control group, and the mice of the inulin-omeprazole group had 29 ± 4% lower *Trpm6* mRNA levels compared to the omeprazole only treated mice (Fig 2C).

The expression of the nongastric H^+^,K^+^-ATPase encoded by *Atp12a* was reduced from 149 ± 12% in the omeprazole-only group down to 58 ± 14% in the inulin-omeprazole group (Fig 2D). In colon and in kidney there were no significant differences present (Fig 2D and 2E).

### Dietary inulin rescues omeprazole-induced low serum Ca^2+^

**Fig 3:**
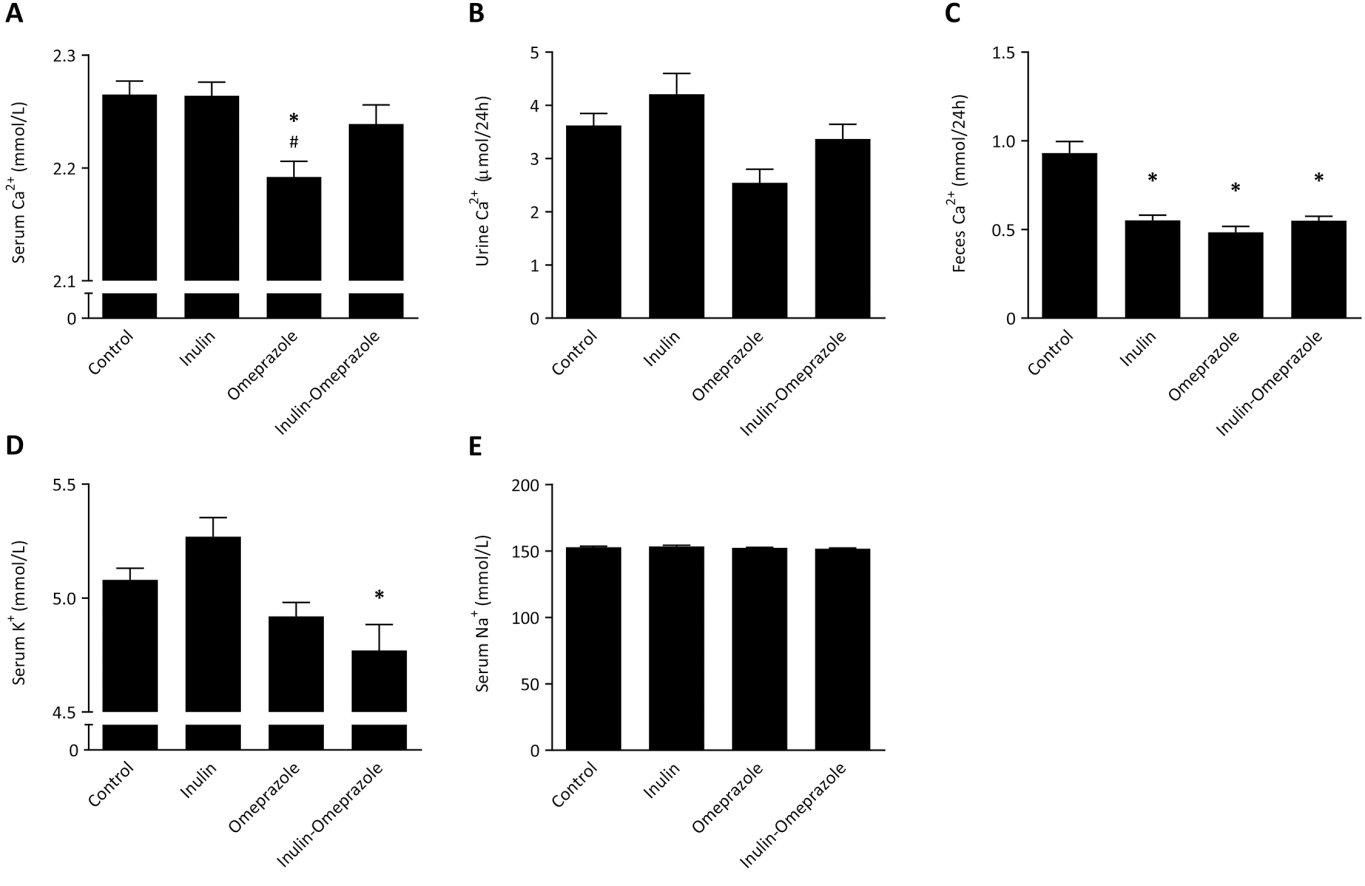
Effects of the combinatory application of omeprazole and 10% inulin enriched diets on electrolytes. Serum Ca^2+^ concentration (A), 24 h urinary Ca^2+^ excretion (B), 24 h fecal Ca^2+^ excretion, serum K^+^ concentration (D) and serum Na^+^ concentration (E). Significant differences compared to control are indicated by * with *P* < 0.05, significant differences between the omeprazole-treated groups are annotated by # with *P* < 0.05. Bars represent means ± SEM, with n = 10 mice per group.

Serum Ca^2+^ of the omeprazole-treated mice was slightly, but, significantly reduced compared to the vehicle-treated control group (2.19 ± 0.01 mmol/L and 2.27 ± 0.01 mmol/L, respectively, *P* < 0.05, Fig 3A). Importantly, application of the inulin diet during omeprazole treatment resulted in a correction of serum Ca^2+^ to the level of control mice and inulin-only treated mice. This is reflected in the 24 h urinary Ca^2+^ excretion; omeprazole treated mice displayed a reduced urinary Ca^2+^ excretion compared to the mice on the inulin diet 2.7 ± 0.3 μmol/24 h and 4.3 ± 0.5 μmol/24 h, respectively, *P* < 0.05 Fig 3B). The fecal excretion of Ca^2+^ in all groups was significantly lower compared to control mice (for inulin 0.55 ± 0.03 mmol/24 h, for omeprazole 0.48 ± 0.03 mmol/24 h, for inulin-omeprazole 0.55 ± 0.03 mmol/24 h vs. control 0.93 ± 0.07 mmol/24 h, Fig 3C).

The serum K^+^ level of the inulin-omeprazole group was significantly lower than that of the control group (4.8 ± 0.1 mmol/L and 5.08 ± 0.05 mmol/L, respectively, P < 0.05, Fig 3D). There were no significant differences in serum Na^+^ levels observed (Fig 3E).

### Inulin prevents PPI-induced upregulation of calciotropic genes

**Fig 4:**
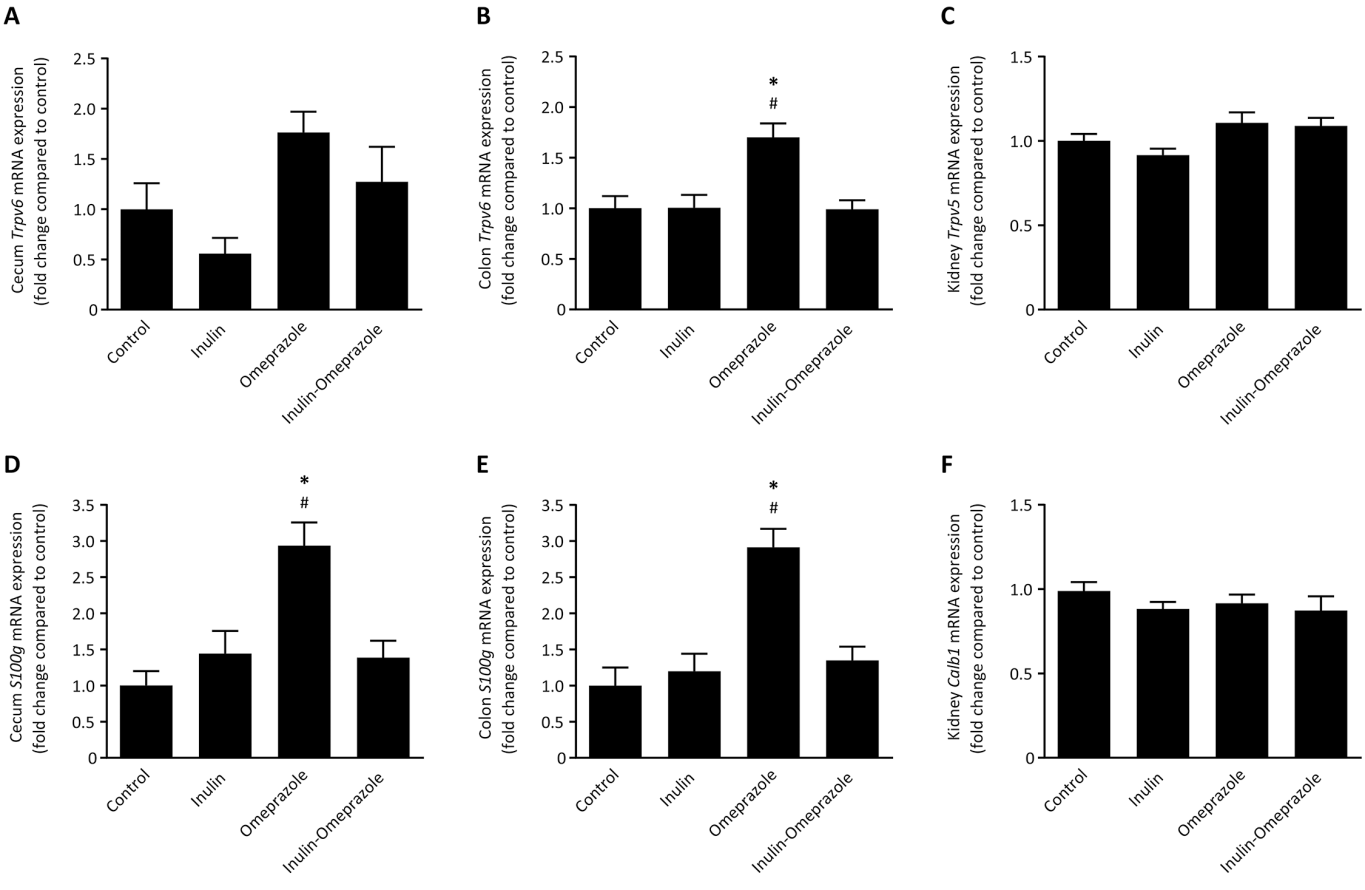
Effects of omeprazole and 10% inulin-enriched diets on mRNA expression levels of the calciotropic genes. *Trpv6* and *S100g* mRNA in cecum (A + D), colon (B + E) and mRNA of *Trpv5* and *Calb1* in the kidney (C + F), corrected for Gapdh and normalized to control. Significant differences compared to control are indicated by * with *P* < 0.05, significant differences between the omeprazole-treated groups are annotated by # with *P* < 0.05. All values represent means ± SEM, with n = 10 mice per group.

In order to investigate if the treatment regimens induced differential gene regulation, the mRNA levels of the main calciotropic genes in the intestine, *Trpv6* together with *S100g* and, *Trpv5* and *Calb1* expressed in the kidney were quantified. In cecum no significant differences were observed for *Trpv6* mRNA levels within the respective diet groups, however in colon *Trpv6* expression was significantly increased to 170 ± 14% compared to the control group (Fig 4A and 4B). Addition of inulin completely abolished this increase. In the kidney *Trpv5* mRNA levels of both omeprazole-treated groups were not significantly different from the respective control groups on normal diets.

In the intestine, the expression pattern of *S100g* was similar in cecum and colon. In both segments, the mRNA levels in omeprazole-only treated mice were significantly increased compared to all the other groups (Fig 4D and 4E). In cecum, *S100g* mRNA levels were increased to 284 ± 32% compared to the control group and in colon to 291 ± 24%. In contrast, no significant differences were observed for the mRNA levels of *Calb1* in the kidney (Fig 4F).

### Dietary inulin enhances short-chain fatty acid production

**Fig 5:**
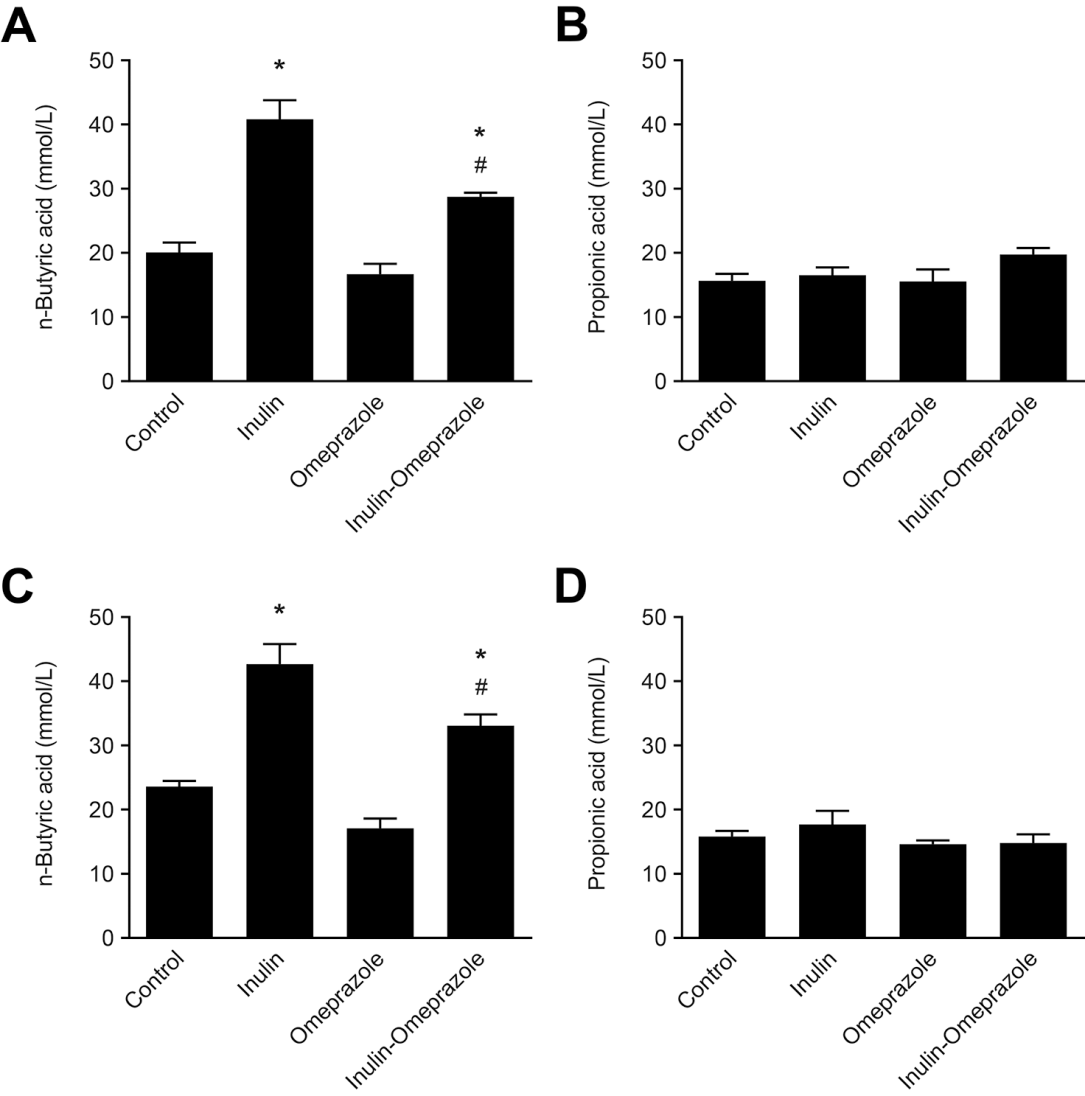
Effects of the combinatory application of omeprazole and inulin-enriched diets on short-chain fatty acid production. Depicted are n-butyric acid (A + C) and propionic acid concentrations (B + D) in cecum (top panels) and colon (bottom panels) in mmol/L. Significant differences compared to the control group are indicated by * with *P* < 0.05, significant differences between the omeprazole-treated groups are annotated by # with *P* < 0.05. Bars represent means ± SEM, with n = 10 mice per group.

To verify whether inulin stimulated intestinal bifidogenic fermentation, SCFA profiles of cecal and colonic contents were determined. In cecum, inulin significantly increased n-butyric acid concentration from 20 ± 2 mmol/L in the control group to 41 ± 3 mmol/L in the inulin group and to 28.7 ± 0.6 mmol/L in the inulin-omeprazole group (*P* < 0.05, Fig 5A). Likewise, in colon inulin significantly increased n-butyric acid concentration from 23.1 ± 0.9 mmol/L in the control group to 43 ± 3 mmol/L in the inulin group and to 33 ± 2 mmol/L in the inulin-omeprazole group (*P* < 0.05, Fig 5C). No differences between the groups were observed for propionic acid in both intestinal segments (Fig 5B and 5D). Moreover, in cecum and colon no differences were observed in the other minor SCFA fractions (with concentrations < 1.5 mmol/L) consisting of iso-propionic acid and iso-butyric acid (data not shown).

## Discussion

This is the first study evaluating a treatment strategy for PPI-induced mineral disturbances in mice. PPI-induced hypocalcemia was counteracted by dietary inulin application. However, a correction of serum Mg^2+^ was not achieved by this approach.

There is a large body of literature showing that dietary inulin fibers stimulate Mg^2+^ and Ca^2+^ absorption in rodents and humans [28, 32]. The most potent stimulation of mineral absorption is achieved by a mixture of long- and short-chain oligofructose-enriched inulin, since certain strains of gut bacteria have a preference for inulin fibers based on chain length [33, 34]. In accordance with several other studies, the inulin product was given at a 10% (w/w) dose added to regular chow [30, 35]. In rats and mice, it has been demonstrated before that at this dose inulin induces luminal acidification in less than one week, enhancing the solubilization of minerals and which subsequently results in osmotic attraction of water in the colon [34, 36]. In the present study a similar effect was demonstrated, since humidification of the feces of the inulin-treated mice was observed, indicating that inulin activated bacterial fermentation also under omeprazole treatment. Indeed, irrespective of the omeprazole treatment n-butyric acid, the main end-metabolite of bifidogenic inulin fermentation, was increased in cecum and colon of the mice on an inulin diet (Fig 5A and Fig 5C) [37, 38]. This result supports previous findings that SCFA, and in particular n-butryic acid, increase Ca^2+^ and Mg^2+^ absorption [27, 39].

The mice on the normal diet showed a significant reduction of serum Ca^2+^ values after 14 days of omeprazole-treatment (Fig 3A). Importantly, concomitant application of dietary inulin prevented this reduction of serum Ca^2+^ values. A pronounced decrease of intestinal Ca^2+^ absorption by PPIs has already been evidenced in early studies and has been confirmed in recent reports [13, 40]. To the authors knowledge, this is the first time that a treatment is successful to prevent PPI-induced Ca^2+^ disturbances.

Although direct measurements of intestinal Ca^2+^ absorption were not performed in this study, the expression profile suggests that intestinal Ca^2+^ transport is affected. In cecum and in colon, omeprazole induced strong increases of intestinal *Trpv6*, together with its Ca^2+^-binding protein encoded by *S100g* in order to correct reduced serum Ca^2+^ levels (Fig 4D and 4E). The addition of inulin completely abolished this increment, which is also reflected in normalized serum Ca^2+^ (Fig 4A). Of note: TRPV6 is the principal epithelial Ca^2+^ channel of the large intestine [41]. It is constitutively open and selective for Ca^2+^ and its expression is highly regulated by the needs for intestinal Ca^2+^ absorption. The absence of differences in renal *Trpv5* and *Calb1* expression and normal urinary Ca^2+^ values indicate that the renal handling of Ca^2+^ was normal in all groups.

Despite the beneficial effect of dietary inulin supplementation on Ca^2+^ absorption, serum Mg^2+^ was still reduced in mice receiving combined omeprazole/inulin treatment. Intestinal Mg^2+^ uptake is mainly facilitated by the epithelial Mg^2+^ channel TRPM6 that is predominantly expressed in cecum and colon [19]. Interestingly, *Trpm6* mRNA expression in cecum was significantly decreased in mice receiving inulin-enriched diets independently of omeprazole treatment (Fig 2A). Given that *Trpm6* mRNA levels are inversely responsive to dietary Mg^2+^ availability, these findings may suggest that dietary inulin stimulated intestinal Mg^2+^ absorption [42]. However, the serum Mg^2+^ level was not restored to normal values.

Urinary Mg^2+^ excretion was increased in mice receiving both inulin and omeprazole compared to mice treated with only omeprazole, which is reflected in a reduced expression of *Trpm6* in the kidney. Although these findings are in line with previous experiments of Rondon and colleagues, reduced renal Mg^2+^ reabsorption is difficult to interpret given the low serum Mg^2+^ values [30]. Because the nephron is devoid from any microbiota, inulin will not directly affect the intratubular pH in the nephron. This is confirmed by the absence of effects of inulin on *Atp12a* expression in the kidney.

Food intake was increased in inulin-omeprazole-treated mice compared to omeprazole-treated mice. However, it is unlikely that this explains the increased serum Ca^2+^ values since serum values of Na^+^, Mg^2+^ and K^+^ were not altered in inulin-omeprazole-treated mice. In our study, serum K^+^ values were reduced in both omeprazole-treated groups, following the same pattern as serum Mg^2+^ values. In the clinic, hypokalemia is often secondary to hypomagnesemia and frequently observed in PPIH patients [5, 8, 12, 13]. The general accepted hypothesis to explain this phenomenon is that hypomagnesemia results in increased K^+^ secretion in the nephron. Given that intracellular Mg^2+^ inhibits the renal ROMK K^+^ channel to reduce renal K^+^ secretion, hypomagnesemia may relieve this inhibition and thus increases K^+^ secretion [43].

The outcomes of our study highlight the need for reissuing the impact of PPIs on Ca^2+^ homeostasis, which recently got neglected by the clinical attention drawn by PPIH. In conclusion, this *in vivo* study provides a treatment perspective for PPI-induced mineral disturbances. Dietary oligofructose enriched inulin fibers prevented the omeprazole-induced reduction of Ca^2+^ absorption and improved intestinal Mg^2+^ absorption in mice. Future clinical studies should investigate whether dietary inulin could prevent PPI-induced mineral deficits in patients.

## Supporting Information

S1 ARRIVE Checklist (PDF)

S1 Table Primer sequences used for real-time PCR analysis. (XLSX)

## References

[1] Metz DC. Managing gastroesophageal reflux disease for the lifetime of the patient: evaluating the long-term options. The American journal of medicine. 2004;117 Suppl 5A:49S–55S. . 15478853 10.1016/j.amjmed.2004.07.009

[2] Sachs G. Proton pump inhibitors and acid-related diseases. Pharmacotherapy. 1997;17(1):22–37. . 9017763

[3] Tjon JA, Pe M, Soscia J, Mahant S. Efficacy and safety of proton pump inhibitors in the management of pediatric gastroesophageal reflux disease. Pharmacotherapy. 2013;33(9):956–71. 10.1002/phar.1299 . 23712734

[4] Patterson Burdsall D, Flores HC, Krueger J, Garretson S, Gorbien MJ, Iacch A, et al Use of proton pump inhibitors with lack of diagnostic indications in 22 Midwestern US skilled nursing facilities. Journal of the American Medical Directors Association. 2013;14(6):429–32. 10.1016/j.jamda.2013.01.021 . 23583000

[5] Hess MW, Hoenderop JG, Bindels RJ, Drenth JP. Systematic review: hypomagnesaemia induced by proton pump inhibition. Alimentary pharmacology & therapeutics. 2012;36(5):405–13. 10.1111/j.1365-2036.2012.05201.x . 22762246

[6] Florentin M, Elisaf MS. Proton pump inhibitor-induced hypomagnesemia: A new challenge. World journal of nephrology. 2012;1(6):151–4. 10.5527/wjn.v1.i6.151 24175253 PMC3782221

[7] Epstein M, McGrath S, Law F. Proton-pump inhibitors and hypomagnesemic hypoparathyroidism. The New England journal of medicine. 2006;355(17):1834–6. 10.1056/NEJMc066308 . 17065651

[8] Hoorn EJ, van der Hoek J, de Man RA, Kuipers EJ, Bolwerk C, Zietse R. A case series of proton pump inhibitor-induced hypomagnesemia. American journal of kidney diseases: the official journal of the National Kidney Foundation. 2010;56(1):112–6. 10.1053/j.ajkd.2009.11.019 . 20189276

[9] Mackay JD, Bladon PT. Hypomagnesaemia due to proton-pump inhibitor therapy: a clinical case series. QJM: monthly journal of the Association of Physicians. 2010;103(6):387–95. 10.1093/qjmed/hcq021 . 20378675

[10] Luk CP, Parsons R, Lee YP, Hughes JD. Proton pump inhibitor-associated hypomagnesemia: what do FDA data tell us? The Annals of pharmacotherapy. 2013;47(6):773–80. 10.1345/aph.1R556 . 23632281

[11] Regolisti G, Cabassi A, Parenti E, Maggiore U, Fiaccadori E. Severe hypomagnesemia during long-term treatment with a proton pump inhibitor. American journal of kidney diseases: the official journal of the National Kidney Foundation. 2010;56(1):168–74. 10.1053/j.ajkd.2010.03.013 . 20493607

[12] Negri AL, Valle EE. Hypomagnesaemia/hypokalemia associated with the use of esomeprazole. Current drug safety. 2011;6(3):204–6. . 22122397 10.2174/157488611797579320

[13] Deroux A, Khouri C, Chabre O, Bouillet L, Casez O. Severe acute neurological symptoms related to proton pump inhibitors induced hypomagnesemia responsible for profound hypoparathyroidism with hypocalcemia. Clinics and research in hepatology and gastroenterology. 2014;38(5):e103–5. 10.1016/j.clinre.2014.03.005 . 24736034

[14] Leontiadis GI, Moayyedi P. Proton pump inhibitors and risk of bone fractures. Current treatment options in gastroenterology. 2014;12(4):414–23. 10.1007/s11938-014-0030-y . 25209137

[15] Thongon N, Krishnamra N. Omeprazole decreases magnesium transport across Caco-2 monolayers. World journal of gastroenterology: WJG. 2011;17(12):1574–83. 10.3748/wjg.v17.i12.1574 21472124 PMC3070129

[16] Thongon N, Krishnamra N. Apical acidity decreases inhibitory effect of omeprazole on Mg(2+) absorption and claudin-7 and -12 expression in Caco-2 monolayers. Experimental & molecular medicine. 2012;44(11):684–93. 10.3858/emm.2012.44.11.077 22940736 PMC3509185

[17] Lameris AL, Hess MW, van Kruijsbergen I, Hoenderop JG, Bindels RJ. Omeprazole enhances the colonic expression of the Mg(2+) transporter TRPM6. Pflugers Archiv: European journal of physiology. 2013;465(11):1613–20. 10.1007/s00424-013-1306-0 . 23756852

[18] de Baaij JH, Hoenderop JG, Bindels RJ. Magnesium in man: implications for health and disease. Physiological reviews. 2015;95(1):1–46. 10.1152/physrev.00012.2014 . 25540137

[19] Groenestege WM, Hoenderop JG, van den Heuvel L, Knoers N, Bindels RJ. The epithelial Mg2+ channel transient receptor potential melastatin 6 is regulated by dietary Mg2+ content and estrogens. Journal of the American Society of Nephrology: JASN. 2006;17(4):1035–43. 10.1681/ASN.2005070700 . 16524949

[20] Voets T, Nilius B, Hoefs S, van der Kemp AW, Droogmans G, Bindels RJ, et al TRPM6 forms the Mg2+ influx channel involved in intestinal and renal Mg2+ absorption. The Journal of biological chemistry. 2004;279(1):19–25. 10.1074/jbc.M311201200 . 14576148

[21] Li M, Du J, Jiang J, Ratzan W, Su LT, Runnels LW, et al Molecular determinants of Mg2+ and Ca2+ permeability and pH sensitivity in TRPM6 and TRPM7. The Journal of biological chemistry. 2007;282(35):25817–30. 10.1074/jbc.M608972200 17599911 PMC3239414

[22] Topala CN, Groenestege WT, Thebault S, van den Berg D, Nilius B, Hoenderop JG, et al Molecular determinants of permeation through the cation channel TRPM6. Cell calcium. 2007;41(6):513–23. 10.1016/j.ceca.2006.10.003 . 17098283

[23] Bai JP, Hausman E, Lionberger R, Zhang X. Modeling and simulation of the effect of proton pump inhibitors on magnesium homeostasis. 1. Oral absorption of magnesium. Molecular pharmaceutics. 2012;9(12):3495–505. 10.1021/mp300323q . 23051182

[24] Petry N, Egli I, Chassard C, Lacroix C, Hurrell R. Inulin modifies the bifidobacteria population, fecal lactate concentration, and fecal pH but does not influence iron absorption in women with low iron status. The American journal of clinical nutrition. 2012;96(2):325–31. 10.3945/ajcn.112.035717 . 22743314

[25] De Vuyst L, Leroy F. Cross-feeding between bifidobacteria and butyrate-producing colon bacteria explains bifdobacterial competitiveness, butyrate production, and gas production. International journal of food microbiology. 2011;149(1):73–80. 10.1016/j.ijfoodmicro.2011.03.003 . 21450362

[26] Scharrer E, Lutz T. Effects of short chain fatty acids and K on absorption of Mg and other cations by the colon and caecum. Zeitschrift fur Ernahrungswissenschaft. 1990;29(3):162–8. . 2251858 10.1007/BF02021554

[27] Leonhard-Marek S, Gabel G, Martens H. Effects of short chain fatty acids and carbon dioxide on magnesium transport across sheep rumen epithelium. Experimental physiology. 1998;83(2):155–64. . 9568475 10.1113/expphysiol.1998.sp004098

[28] Coudray C, Demigne C, Rayssiguier Y. Effects of dietary fibers on magnesium absorption in animals and humans. The Journal of nutrition. 2003;133(1):1–4. . 12514257 10.1093/jn/133.1.1

[29] Coudray C, Feillet-Coudray C, Tressol JC, Gueux E, Thien S, Jaffrelo L, et al Stimulatory effect of inulin on intestinal absorption of calcium and magnesium in rats is modulated by dietary calcium intakes short- and long-term balance studies. European journal of nutrition. 2005;44(5):293–302. 10.1007/s00394-004-0526-7 . 15340751

[30] Rondon LJ, Rayssiguier Y, Mazur A. Dietary inulin in mice stimulates Mg2+ absorption and modulates TRPM6 and TRPM7 expression in large intestine and kidney. Magnesium research: official organ of the International Society for the Development of Research on Magnesium. 2008;21(4):224–31. . 19271420

[31] Livak KJ, Schmittgen TD. Analysis of relative gene expression data using real-time quantitative PCR and the 2(-Delta Delta C(T)) Method. Methods. 2001;25(4):402–8. 10.1006/meth.2001.1262 . 11846609

[32] Holloway L, Moynihan S, Abrams SA, Kent K, Hsu AR, Friedlander AL. Effects of oligofructose-enriched inulin on intestinal absorption of calcium and magnesium and bone turnover markers in postmenopausal women. The British journal of nutrition. 2007;97(2):365–72. 10.1017/S000711450733674X . 17298707

[33] Gibson GR, Beatty ER, Wang X, Cummings JH. Selective stimulation of bifidobacteria in the human colon by oligofructose and inulin. Gastroenterology. 1995;108(4):975–82. . 7698613 10.1016/0016-5085(95)90192-2

[34] Coudray C, Tressol JC, Gueux E, Rayssiguier Y. Effects of inulin-type fructans of different chain length and type of branching on intestinal absorption and balance of calcium and magnesium in rats. European journal of nutrition. 2003;42(2):91–8. 10.1007/s00394-003-0390-x . 12638030

[35] Roberfroid MB. Introducing inulin-type fructans. The British journal of nutrition. 2005;93 Suppl 1:S13–25. . 15877886 10.1079/bjn20041350

[36] Geeta Shukla AV, Jashandeep Singh and Hariom Yadav. Prebiotic Inulin Alters the Colonic Mass, pH, Microflora and Short Chain Fatty Acids in 1,2-Dimethylhydrazine Dihydrochloride Induced Early Colon Carcinogenesis in Male Laca Mice. J Prob Health. 2014;2:121 10.4172/2329-8901.1000121

[37] Rossi M, Corradini C, Amaretti A, Nicolini M, Pompei A, Zanoni S, et al Fermentation of fructooligosaccharides and inulin by bifidobacteria: a comparative study of pure and fecal cultures. Applied and environmental microbiology. 2005;71(10):6150–8. 16204533 10.1128/AEM.71.10.6150-6158.2005 PMC1265942

[38] Jung TH, Jeon WM, Han KS. In Vitro Effects of Dietary Inulin on Human Fecal Microbiota and Butyrate Production. Journal of microbiology and biotechnology. 2015 . 26095388 10.4014/jmb.1505.05078

[39] Lutz T, Scharrer E. Effect of short-chain fatty acids on calcium absorption by the rat colon. Experimental physiology. 1991;76(4):615–8. Epub 1991/07/01. . 1910768 10.1113/expphysiol.1991.sp003530

[40] Mizunashi K, Furukawa Y, Katano K, Abe K. Effect of omeprazole, an inhibitor of H+,K(+)-ATPase, on bone resorption in humans. Calcified tissue international. 1993;53(1):21–5. . 8102318 10.1007/BF01352010

[41] van de Graaf SF, Hoenderop JG, Bindels RJ. Regulation of TRPV5 and TRPV6 by associated proteins. American journal of physiology Renal physiology. 2006;290(6):F1295–302. 10.1152/ajprenal.00443.2005 . 16682485

[42] de Baaij JH, Groot Koerkamp MJ, Lavrijsen M, van Zeeland F, Meijer H, Holstege FC, et al Elucidation of the distal convoluted tubule transcriptome identifies new candidate genes involved in renal Mg(2+) handling. American journal of physiology Renal physiology. 2013;305(11):F1563–73. 10.1152/ajprenal.00322.2013 . 24089412

[43] Huang CL, Kuo E. Mechanism of hypokalemia in magnesium deficiency. Journal of the American Society of Nephrology: JASN. 2007;18(10):2649–52. 10.1681/ASN.2007070792 . 17804670

